# Clinico- pathologic presentation of hypersensitivity pneumonitis in Egyptian patients: a multidisciplinary study

**DOI:** 10.1186/s40248-017-0091-6

**Published:** 2017-05-08

**Authors:** Dalia Abd El-Kareem, Yosri M. Akl, Gina A. Nakhla, Ali A. Elhindawi, Mahmoud A. Eltorky

**Affiliations:** 1 Department of Pathology, Faculty of Medicine – Kasr Al-Ainy, Cairo University, Cairo, 11562 Egypt; 2 Department of Pulmonary Medicine, Faculty of Medicine – Kasr Al-Ainy, Cairo University, Cairo, 11562 Egypt; 3 Department of Pathology, University of Texas Medical Branch (UTMB), 301 University Blvd #1, Galveston, TX 77555 USA

**Keywords:** Hypersensitivity pneumonitis, Diffuse parenchymal lung diseases, Interstitial lung diseases, Multidisciplinary approach

## Abstract

**Background:**

Hypersensitivity pneumonitis (HP) is a common diffuse parenchymal lung disease in Egypt which can be difficult to recognize due to the dynamic symptoms & associated environmental factors.

**Methods:**

Forty-three Egyptian patients were enrolled in this study, presenting with dyspnea and cough, predominant ground-glass opacity (GGO) in high-resolution computed tomography (HRCT) where lung biopsy was needed to establish the diagnosis.

**Results:**

The age range was 15 to 60 years. Females represented 90.7% (39 patients) while 9.3% (4 patients) of our patients were males. History of contact with birds was detected in 9 (20.9%) patients. Most of our patients (60.5%) didn’t have exposure history, and only 8 patients (18.6%) were living in geographic areas in Egypt that are known for the exposure to environmental etiologic factors (cane sugar exhaust fumes). The most common HRCT pattern was GGO with mosaic parenchyma in 18 patients (41.86%), followed by GGO with centrilobular nodules in 9 patients (20.93%), then isolated diffuse GGO in 5 patients (11.62%), GGO with traction bronchiectasis in 4 patients (9.3%), GGO with consolidation in 3 patients (6.97%), GGO with reticulations in 2 patients (4.65%), and GGO with cysts in 2 patients (4.65%). The most common histologic finding was isolated multinucleated giant cells in 38 patients (88.3%) commonly found in airspaces (24 patients) and less commonly in the interstitium (14 patients), followed by interstitial pneumonia and cellular bronchiolitis in 36 patients (83.7% each), interstitial ill-formed non-necrotizing granulomas in 12 patients (27.9%), fibrosis in 10 patients (23.2%), and organizing pneumonia pattern in 4 patients (9.3%).

**Conclusion:**

The diagnosis of HP presenting with predominant GGO pattern in HRCT requires a close interaction among clinicians, radiologists, and pathologists. Some environmental and household factors may be underestimated as etiologic factors. Further environmental and genetic studies are needed especially in patients with negative exposure history.

## Background

Hypersensitivity pneumonitis (HP) is an increasingly recognized, immunologically mediated form of diffuse parenchymal lung disease that is usually caused by the exposure to various types of inhaled antigens [1, 2].

HP can sometimes be difficult to recognize because the manifestations of HP are dynamic and associated with complex environmental and host factors. The initial diagnostic evaluation for any patient presenting with cough or dyspnea should include detailed environmental and occupational histories. Some additional evidence for HP may include serology, chest imaging, laboratory-based antigen inhalation challenges, bronchioloalveolar lavage (BAL), and if the diagnosis is still uncertain, lung biopsy is required [3].

This disease passes through 3 phases: acute, subacute and chronic [4].

Conventional radiology is not of great value, especially in patients with mild acute and subacute forms of HP [4, 5]. The sensitivity of high-resolution computed tomography (HRCT) for the detection of HP is greater than that of chest radiography [6].

Lung biopsy may play a critical role in separating chronic hypersensitivity pneumonia from other forms of diffuse parenchymal lung diseases, especially in patients with no specific antigenic exposure [7] or in patients with atypical clinical/radiological presentations [8].

## Objectives

The aim of this study is to correlate the clinical presentation, radiologic features and histopathologic criteria of hypersensitivity pneumonitis, together with encouraging the multidisciplinary approach in the diagnosis of DPLDs.

## Methods

This study included 43 Egyptian patients, presented predominantly with ground glass opacity (GGO) in high-resolution computed tomography (HRCT) where the diagnosis was not established with the clinical features and the HRCT alone and a lung biopsy was the decision taken by the multidisciplinary team.

Patients were enrolled from Department of Pulmonary Medicine, Cairo University hospitals — Kasr Al-Ainy, Cairo, Egypt. They have had a history of dyspnea and dry cough for 6 weeks or more.

Medical thoracoscopy was used for obtaining lung biopsy in 23 patients, whereas transbronchial lung biopsy (TBLBs) in 15 patients, and video- assisted thoracoscopic surgery (VATS) in 5 patients.

All clinical and radiologic data together with a full occupational, exposure, smoking, family and drug history were collected. Lung biopsies were examined in Department of Pathology, Cairo University— Kasr Al-Ainy, Cairo, Egypt.

Medical thoracoscopic and VATS biopsies were injected by 10% neutral buffered formalin for re-inflation and fixation, serially sectioned and all specimens were embedded in paraffin blocks. Sections of 3 μm thickness were cut from the formalin fixed paraffin blocks on multiple levels and all slides were stained manually by hematoxylin and eosin (H&E) and Masson’s Trichrome stains.

After the histologic diagnosis was established, the final diagnosis was concluded in a multidisciplinary manner with a correlation of clinical, radiologic, and pathologic findings.

## Results

This study included 43 Egyptian patients presenting clinically with dry cough and dyspnea, HRCT findings revealed predominant ground glass opacity associated with other patterns and were diagnosed by the multidisciplinary team after lung biopsy as hypersensitivity pneumonitis.

The age range in our patients was 15 to 60 years with a significant female predominance as 90.7%; in fact 39 patients were females and only 9.3% (4 patients) were males.

Nine (20.9%) patients had a history of contact with birds (pigeons) and eight patients (18.6%) were living in those geographic areas in Egypt known for the heavy exposure to cane sugar exhaust fumes, while most of the patients (60.4%) didn’t have any history of exposure to the known antigens.

**Fig. 1:**
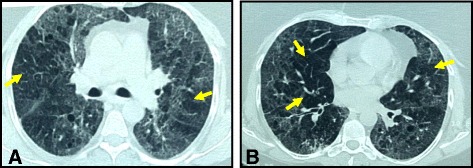
HRCT of HP in a patient: (**a**) and (**b**) showing bilateral ground glass opacity with areas of air trapping (mosaic parenchyma) (*arrows*)

**Fig. 2:**
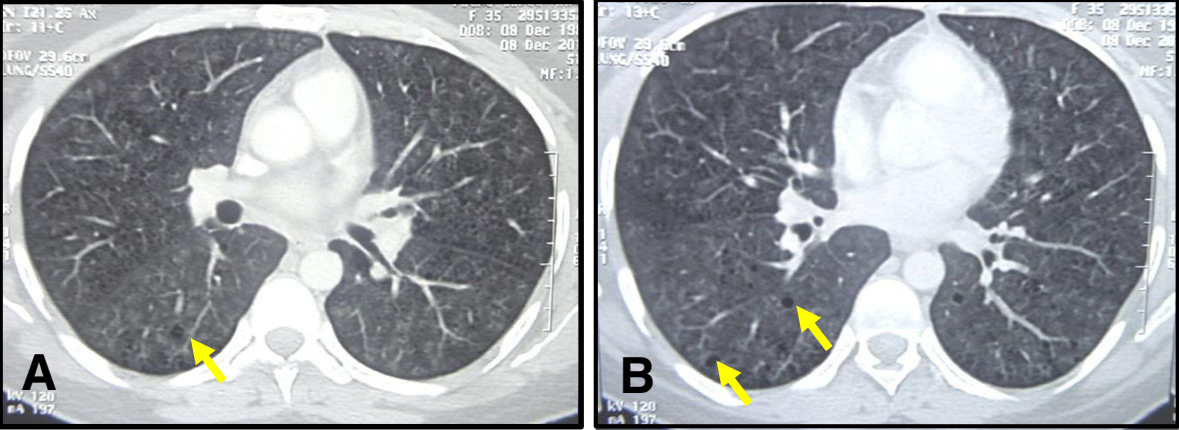
HRCT of HP patient (**a**) and (**b**) showing predominant ground glass opacity with centrilobular nodules and scattered small sized cysts (*arrows*)

The most common HRCT pattern in our patients was GGO with air trapping (Mosaic parenchyma) in 18 patients (41.8%) (Fig. 1). The second common pattern was GGO in association with centrilobular nodules in nine patients (20.9%) (Fig. 2), followed by the isolated diffuse GGO in five patients (11.6%), then the GGO with traction bronchiectasis in four patients (9.3%), GGO with areas of consolidation in three patients (6.9%), GGO with reticulations in two patients (4.6%), and lastly GGO with cysts in two patients (4.6%) Table (1).

**Table 1:** HRCT findings in correlation with histologic features

Number of patients (%)	HRCT	Histopathology
18 (41.8%)	GGO^a^ + Air trapping (mosaic) (Fig. 1)	Mild alveolar septal expansion with lymphoplasmacytic cellular inflammatory infiltrate with peribronchiolar accentuation and evident cellular/granulomatous bronchiolitis
9 (20.9%)	GGO + Centrilobular Nodules (Fig. 2)	Interstitial pneumonia with interstitial and peribronchiolar ill-formed non-necrotizing granulomas
5 (11.6%)	Diffuse isolated GGO	Alveolar septal expansion with lymphoplasmacytic cellular inflammatory infiltrate with or without interstitial fibrosis
4 (9.3%)	GGO + Traction bronchiectasis	Alveolar septal expansion with lymphoplasmacytic cellular inflammatory infiltrates with peribronchiolar accentuation and cellular bronchiolitis with variable degrees of interstitial, subpleural or peribronchiolar fibrosis with or without remodeling.
3 (6.9%)	GGO + Consolidation	Alveolar septal expansion with lymphoplasmacytic cellular inflammatory infiltrate
2 (4.6%)	GGO + Reticulations	Alveolar septal expansion with lymphoplasmacytic cellular inflammatory infiltrate
2 (4.6%)	GGO + Cysts	Alveolar septal expansion with lymphoplasmacytic cellular inflammatory infiltrate with airspace dilatation

^a^GGO: Ground glass opacity

**Fig. 3:**
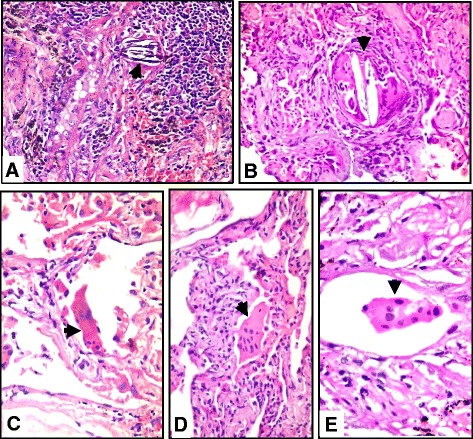
Photomicrographs showing isolated multinucleated giant cells (*arrowheads*) from different HP cases, seen in the interstitium in (**a**) and (**b**) and inside the air spaces (**c**),(**d**) and (**e**). Contains calcium phosphate inclusion in (**a**) and cholesterol clefts in (**b**). [H&E, Original magnifications x200, x400, x400, x400 and x1000, **a** through **d**, respectively]

**Fig. 4:**
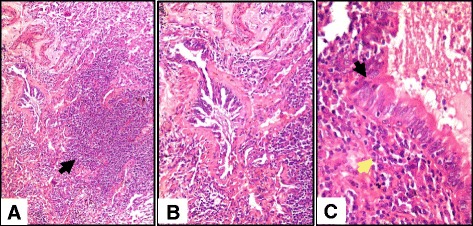
Lung biopsy of the same HP patient in Fig. 1 (Photomicrographs) showing (**a**) Dense interstitial peribronchiolar inflammatory infiltrates (*black arrowhead*) [H&E original magnification x40], (**b**) Peribronchiolar lymphoplasmacytic cellular infiltrates (cellular bronchiolitis) and fibrosis [H&E original magnification x100] (**c**) Higher magnification showing the inflammatory cells (*yellow arrowhead*) in the bronchiolar wall and lining epithelium (*black arrowhead*) [H&E original magnification × 200]

**Fig. 5:**
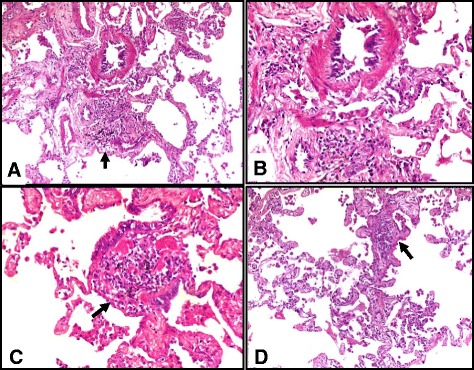
Photomicrographs showing interstitial and peribronchiolar ill- formed non-necrotizing granulomas (*arrows*) together with interstitial lymphoplasmacytic inflammatory infiltrates. [H&E original magnification × 40, ×100, ×100 and × 40 in **a** through **d**, respectively]

The most common histological finding observed in our patients was the presence of isolated multinucleated giant cells (IMNGCs) in 38 patients (88.3%). In 24 (55.8%) of them, the IMNGCs were observed inside the air spaces, whereas in the interstitium in 14 (32.5%) of them (Fig. 3). This finding was followed in frequency by the presence of both interstitial pneumonia and cellular bronchiolitis in 36 patients (83.7%) (Fig. 4). The interstitial pneumonia was distributed as diffuse interstitial pneumonia with peribronchiolar accentuation of the inflammation (centrilobular) in 17 patients (39.5%), diffuse interstitial pneumonia in 11 patients (25.5%) and the inflammation was only centrilobular in 8 patients (18.6%). Cellular bronchiolitis was formed of lymphoplasmacytic infiltrates in 32 patients (74.4%), this was in addition to histiocytes, multinucleated giant cells and poorly formed granulomas in the wall of the bronchioles in 4 patients (9.3%). The interstitial ill-formed non-necrotizing granulomas were seen in 12 patients (27.9%) (Fig. 5). Fibrosis was found in 10 patients (23.2%) and we further subclassified it into alveolar septal fibrosis (interstitial/NSIP-like) in four patients (9.3%), peribronchiolar fibrosis in three patients (6.9%) or peripheral and subpleural with remodeling (UIP-like) in two patients (4.6%). Organizing pneumonia pattern was seen in four patients (9.3%) Table (2).

**Table 2:** A detailed histopathological features of HP cases

Histological Features	n (%)		*n* = 43 (%)
Interstitial pneumonia	36 (83.7%)	Centrilobular	8 (18.6%)
		Diffuse	11 (25.5%)
		Centrilobular and diffuse	17 (39.5%)
Cellular bronchiolitis (Fig. 4)	36 (83.7%)	Lymphoplasmacytic	32 (74.4%)
		Histiocytes and multinucleated giant cells	4 (9.3%)
Interstitial granulomas (Fig. 5)	12 (27.9%)		
Isolated Multinucleated Giant Cells (IMNGCs) (Fig. 3)	38 (88.3%)	Interstitial	14 (32.5%)
		Air spaces	24 (55.8%)
Organizing Pneumonia (OP)	4 (9.3%)		
Fibrosis	10 (23.2%)	Interstitial (NSIP^a^- like)	4 (9.3%)
		Peribronchiolar	3 (6.9%)
		UIP^a^-like	2 (4.6%)

^a^NSIP: Non specific interstitial pneumonia, UIP: Usual interstitial pneumonia

The impact of the multidisciplinary discussion (MDD) approach was reflected in many patients. Twenty-two (51.1%) of our patients were initially suspected as HP on clinical basis and this diagnosis was confirmed afterradiology and lung biopsyby the multidisciplinary discussion approach. On the other hand, six patients were suspected to be HP on clinical and radiologic basis but after lung biopsy and multidisciplinary discussion this diagnosis was not confirmed and the final diagnosis was changed to sarcoidosis, non-specific interstitial pneumonia (NSIP) or interstitial pathology due to infective etiology (these patients were excluded from the study) (Table 3).

**Table 3:** Correlation between clinic-radiologic suspicion and the final diagnosis reached after MDD

Clinico-Radiologic Diagnosis	Number of patients	Final Diagnosis (MDD)^a^
HP^a^	22	HP
	3	NSIP^a^
	1	Infection
	2	Sarcoidosis
Organizing Pneumonia	2	HP
PAP^a^	2	
Opportunistic Infection	3	
Malignant^b^	4	
Sarcoidosis	4	HP
NSIP	6	HP

^a^MDD: Multidisciplinary Discussion, HP: Hypersensitivity Pneumonitis, NSIP: Nonspecific interstitial pneumonia, PAP: Pulmonary alveolar proteinosis

^b^Malignant: adenocarcinoma (lepidic form/formerly bronchioloalveolar carcinoma)

Twenty-one patients (48.8%) were presenting with dyspnea and cough and radiological GGO was the predominant pattern in HRCT. Their provisional diagnoses included organizing pneumonia (two patients), pulmonary alveolar proteinosis (PAP) (two patients), opportunistic lung infections (three patients), non-specific interstitial pneumonia (NSIP) (six patients), sarcoidosis (four patients) or malignant (four patients). These patients’ lung biopsy and MDD confirmed the diagnosis of HP (Table 3).

## Discussion

Considering the exposure to potentially offending antigens as a cause for hypersensitivity pneumonitis, nine (20.9%) out of our 43 patients had a history of breeding birds and eight (18.6%) were living in the known areas of exposure to the exhaust of cane sugar factories in Egypt as Kom-Ombo and Elhawamdeya. The most common causes of HP are avian antigens from pigeons, parakeets, budgerigars, and parrots (due to occupational, recreational, or domestic exposure), with a documented rate of “bird fancier’s lung” of up to 21% [9, 10].

Although many authors stated that the antigenic exposure to the known antigens was present in most of their patients [11, 12], we strikingly observed that most of our patients (60.4%) didn’t have any history of exposure to a known antigen.

We suggest that some domestic factors, such as indoor (household) molds, dust, together with the heavy air pollution in our country, seem to be under-recognized as etiologic factors for HP, and further investigations of this point may explain the large number of patients with no history of exposure to the commonly known antigens [13].

The pathogenesis of hypersensitivity pneumonitis was proposed in the literature by the repeated inhalation of specific antigens from environmental exposure in sensitized individuals, which develops as a result of a cell-mediated immune response in the lung [14]. That is why the mainstay of diagnosis is a precise exposure history and the main management is through avoidance of exposure to the causative agent, but it is still believed that HP is not an atopic disease and is not associated with increased IgE level or peripheral eosinophilic count [15].

Cough and dyspnea are the chief presenting symptoms of HP. [11] Smoking history and its relation to HP is still not clear, as it was negative in all our patients, but a positive smoking history in HP patients was reported in the literature in ex-smoker patients [11].

The predominance of GGO in HRCT in our patients and in the literature is classic in HP but it is a non-specific finding [11]. Patchy or diffuse GGO can be seen in acute, subacute, and chronic stages of HP [16].

The most common HRCT pattern in association with GGO is the presence of air trapping (Mosaic parenchyma) [17]. The presence of air trapping has a high specificity and sensitivity for hypersensitivity pneumonitis, which was observed in 18 of our patients (41.8%).

The prevalence of GGO in association with centrilobular nodules was also high in our patients (20.9%). Some authors reported a high frequency of reticular opacities in their HP patients (65%) while this was seen in a trivial portion (4.6%) of our patients [11].

The histologic features of HP depend on the stage of the disease [16]. Biopsies are usually performed during the subacute phase, when changes of chronic interstitial pneumonia predominate with thickening of the alveolar walls and alveolar septa, while the acute stage is rarely biopsied [12].

The histologic features in HP are cellular bronchiolitis (Lymphoplasmacytic), interstitial pneumonia, isolated multinucleated giant cells (MNGCs), organizing pneumonia (OP), poorly formed granulomas, and interstitial fibrosis [11].

The histopathologic changes of HP have been reported by several investigators [9, 10, 18, 19], giving rise to the classic triad of cellular bronchiolitis, interstitial pneumonia, and poorly formed non-necrotizing granulomas [20].

Isolated multinucleated giant cells (IMNGCs) was the most frequent histologic feature in our study (88.3%). They occur frequently in hypersensitivity pneumonitis, and in some cases may represent a striking feature. Giant cells often contain various nonspecific cytoplasmic inclusions such as Schaumann bodies, asteroid bodies, cholesterol clefts, and birefringent calcium salts. The IMNGCs were found in the peribronchiolar interstitium and in the terminal airways in many of our cases [12, 13, 20, 25, 26].

IMNGCSs can be seen inside the air spaces, which was the most frequent site observed in our cases (55.8%), but can also be seen in the interstitium. Its presence within the pleura was also rep﻿orted in literature [11].

The pathology literature and major pulmonary pathology textbooks suggest that the granulomatous inflammation in HP should be located in the pulmonary interstitium [1, 19, 27–29], and they describe the granulomas and MNGCs in HP as being present within the peribronchiolar stroma or alveolar septa, excluding the airspaces [9, 18, 19].

However, we found that granulomas and IMNGCs are not restricted to the pulmonary interstitium only [11].

Feary & Szram, stated this as a feature not previously noted and became a source of confusion in the literature and in teaching about HP, which may lead pathologists to exclude HP from the differential diagnosis [12].

It was previously stated that when the IMNGCs are present, they are accompanied by poorly formed interstitial non-necrotizing granulomas [12], but we noticed in our cases that the IMNGCs are not linked to the interstitial granulomas and that it’s not a must to coexist in the same case or at least in the same biopsy and it may need a second biopsy site to confirm such observation.

Chronic interstitial pneumonia is the most frequently encountered histologic lesions in HP [11, 21, 22] detected in 83% of our patients and this percent has reached 95% in some studies [11].

The interstitial inflammation in our patients was distributed as centrilobular in 8 patients (18.6%); diffuse in 11 patients (25.5%) and both centrilobular and diffuse in 17 patients (39.5%).

The associated cellular infiltrate in the wall of the alveoli and small airways (alveolitis and cellular bronchiolitis) was predominated by lymphocytes, plasma cells, multinucleated giant cells and only occasional eosinophils and neutrophils [20]. Poorly formed non-necrotizing granulomas can also be seen in some cases. Well-formed granulomas are only seen in hot tub lungs [20].

Cellular bronchiolitis is also one of the classic features of HP, and Hypersensitivity pneumonitis usually comes in the differential diagnosis of cellular bronchiolitis, whether there are fibrotic changes or not [23]. It is usually predominating in HP cases as reported by many authors [11]. This was seen in 83.7% (36) of our patients, 74.4% (32 patients) of them showed lymphoplasmacytic infiltration of the airways together with some lymphoid aggregates and 9.3% (four patients) of them showed non- necrotizing granulomatous forms of bronchiolitis. While hypersensitivity pneumonitis (HP) is theoretically in the differential diagnosis of a lymphoid interstitial pneumonia (LIP) pattern, the intensity of lymphoid infiltration is much less in HP and the infiltrates are usually airway-centered even in the presence of airspace dilatation and cyst formation [24].

Collections of foamy alveolar macrophages may be seen in some cases filling the peribronchiolar air spaces, representing a microscopic feature of obstructive pneumonia and indicating the presence of the previously mentioned bronchiolitis which results in small airway dysfunction [20].

Poorly formed interstitial and peribronchiolar non-necrotizing granulomas are usually the commonest histologic features of HP, and even pleural granulomas [11]. Also, airspace granulomas were described by many authors in as high frequency as 85% and sometimes it was the only site for the granulomas [11, 12]. In fact, the frequency of the poorly formed non-necrotizing granulomas was relatively low in our patients, only in 27.9% of them being seen interstitial and peribronchiolar. Airspace and pleural granulomas were never observed in our study.

It was reported in the literature that most of the HP patients whose lung biopsy showed airspace granulomas had a positive exposure history (parakeet, peacock, household mold, cockatiel/continuous positive airway pressure device (CPAP) humidifier, and moldy hay), and rarely positive serology for Thermoactinomyces Vulgaris and avian antigens in connection to chicken coop exposure [11].

The recognition that granulomas and isolated MNGCs can be isolated to the airspaces will undoubtedly assist in properly identifying additional HP cases that may have previously escaped observation [11].

Organizing pneumonia pattern (OP) can be associated with HP in variable frequencies, ranging from 85% [11] to as low as 13.9% in our study and other similar studies [10, 19, 29].

The effect of steroids and other anti-inflammatory medications received by our patients prior to the biopsy may be responsible for this big variation in the frequency of OP.

We didn’t report any kind of pleural affection in our thoracoscopic biopsied patients, although it was noted in the literature either chronic pleural inflammation, granulomas or IMNGCs, but the pleural affection was never reported in isolation, and interstitial inflammation was always a constant reported finding [11].

Pulmonary fibrosis in HP patients evolves with time, with variable distribution and the frequency of its detection depends on the timing of the disease diagnosis. It is observed in 10 of our patients (23.2%) and subclassified as interstitial (alveolar septal/NSIP-like) in four patients (9.3%), peribronchiolar (bronchiolocentric) in three patients (6.9%) and peripheral, patchy and subpleural (UIP- like) in two patients (4.6%) [11, 13].

When HP reaches the advanced chronic stage, fibrosis and architectural distortion that mimic the radiological and histological features of UIP will be seen [21, 30–32], in the form of histological patchy fibrosis, peripheral subpleural honeycombing, and fibroblastic foci. A difference is that honeycombing has a predominantly upper lobe distribution only in patients with hypersensitivity pneumonitis [33].

Also lung biopsies from patients with well-established clinical diagnoses of hypersensitivity pneumonitis may show histologic overlap with nonspecific interstitial pneumonia (NSIP) [20]. This occurs in the form of chronic interstitial pneumonia with relatively uniform alveolar septal expansion by chronic inflammatory cells with or without fibrosis [34].

When cellular, granulomatous bronchiolitis is found away from the areas of fibrosis, this is more with HP than UIP. Also peribronchiolar metaplasia occurs more frequently in HP compared with UIP/IPF, but it is common in both and therefore cannot be used by itself as a discriminating histological feature [33, 35]. The presence of peribronchiolar fibrosis in HP cases with UIP-like fibrosis is a useful feature to distinguish chronic HP from idiopathic UIP [30, 31, 33, 35].

Ultimately, the diagnosis of HP remains the one made in concert with clinical, serological, microbiological, and imaging correlation. The histopathologic findings can prove helpful, especially in cases where an exposure is not apparent as in most of our patients; the recognition of HP may lead to increased identification of inciting antigens, and has been linked to improved survival rate in chronic HP [36].

## Conclusion

- Definite diagnosis sometimes can’t be accurately established by clinical or radiological features alone in HP and lung biopsy is needed to establish a final diagnosis.
- Lung biopsy and MDD will make the diagnosis easily reached and this will provide an earlier diagnosis for these patients and hence the earlier start of treatment, better response to therapy and better prognosis.
- Although lung biopsy can be the ultimate procedure to reach the diagnosis in difficult cases, the integration of pathological, radiological and clinical data of the patient are vital for the correct diagnosis and management.
- The diagnostic approach to HP and other DPLD, especially those presenting with predominant GGO pattern in HRCT, requires a close interaction between Clinicians, Radiologists, and Pathologists.

## Abbreviations

BAL: Bronchioloalveolar lavage
CPAP: Continuous positive airway pressure
DPLD: Diffuse parenchymal lung diseases
GGO: Ground glass opacity
HP: Hypersensitivity pneumonitis
HRCT: High resolution computed tomography
IgE: Immunoglobulin E
IMNGCs: Isolated multinucleated giant cells
IPF: Idiopathic pulmonary fibrosis
LIP: Lymphocytic interstitial pneumonia
MDD: Multidisciplinary discussion
NSIP: Non specific interstitial pneumonia
OP: Organizing pneumonia
PAP: Pulmonary alveolar proteinosis
TBLB: Transbronchial lung biopsy
UIP: Usual interstitial pneumonia
VATS: Video-assisted thoracoscopic surgery
